# The Treatment of Typhoid Fever

**Published:** 1882-04

**Authors:** 


### Selections

### The Treatment of Typhoid Fever.

We copy entire the proceedings of the Boston Society for Medical Improvement, from the *Boston Medical and Surgical Journal* of Feb. 23.

Dr. A. L. Mason opened the debate with the following remarks:

Having been requested by the President of the Society to say a few words by way of opening a more general discussion of the management of typhoid fever, I will briefly call your attention to the different classes of cases, as ordinarily met with, and to their varying therapeutic requirements. These latter, in a general way, are: 1, Ventilation and sunlight. 2, The management of the diet and bowels. 3, The use of stimulants and other remedies. 4, The regulation of the temperature. 5, The disinfection of the stools.

It appears from hospital record books that about seventy-five per cent. of the cases do well if proper attention is given to the points mentioned under the first two headings, with such mild measures as may be necessary for securing sleep and allaying discomfort; that is, under an expectant plan of treatment.

A diet consisting mainly of milk, with plenty of cold water to drink, probably meets the requirements better than any other, and as a rule two quarts of milk will supply sufficient nutriment. Patients will sometimes manage three and four quarts, but it is often found undigested and sour in the stools.

In cases which are prolonged to five or six weeks, without relapse, with slightly raised temperature and quick pulse, from muscular debility, too long continuance of liquid diet is apt to delay recovery. On the other hand, too early ingestion of solids, before the intestinal ulcers are well advanced toward healing, may cause almost immediate aggravation of the symptoms. Examination of the abdomen sometimes enables us to determine whether the unhealed lesions or whether muscular degeneration, with nervous exhaustion, are responsible for the tardy convalescence.

With regard to the bowels, it has been an open question whether any interference is desirable beyond checking too free a diarrhœa. Cases which are costive throughout often do perfectly well, and the absence of peristaltic action during the second and third weeks would be expected to contribute to the healing of the ulcers. But in ordinary cases, with a tendency to constipation, it is better to empty the rectum occasionally by enemata. Few practitioners care to meddle with the bowels any further than that, although the use of occasional laxatives throughout the disease has been said to be of service in relieving the abdominal discomfort and tympanites.

An exception may be made in favor of the German mode of giving ten grains of calomel at the outset, a practice which has appeared to me to give the patient a fairer start, so to speak, than allowing the bowels to remain overloaded. There is probably no greater virtue in calomel than in a dose of castor oil or other cathartics, except that it is easy to administer.

*The use of stimulants and other remedies.* As was before remarked, about seventy-five per cent. of the cases are mild and get well under purely expectant treatment, perhaps requiring a little alcohol towards the close to hasten convalescence. But of the remaining twenty-five per cent. of cases about ten per cent. assume a severer type, and, after suffering from the graver symptoms caused by the higher range of temperature, the deeper intestinal lesions, and the ataxic state, perhaps after having survived hæmorrhages from the bowels, bed sores, and an extreme degree of muscular degeneration, these cases also, to some extent by virtue of the rational therapeutics of to-day, rally from the last stages of physical exhaustion and recover.

And, lastly, we have the fatal cases to deal with, varying in number from five to twenty-five per cent., according to the severity of the epidemic, and, if we can draw any conclusions from statistics, according also to the therapeutic measures which are adopted.

Among the requisites for successfully tiding over many critical cases alcohol stands first, a reserve force, however, which should not be wasted by too early use. During the last autumn at the City Hospital, Dr. Stedman adopted the plan, which I continued, of withholding stimulants from all cases in which the pulse did not rise to 120, that is, in the majority of cases, unless there was some special indication for their use,—such as a weak first-sound of the heart, an irregular or dicrotic pulse. These cases did well, and, in fact, the death-rate this year was low, about eight or nine per cent. The epidemic, although extensive, was not severe.

Other drugs which have been useful are, ergot in ataxic cases, with capillary stasis from a tendency to vaso-motor paralysis; the mineral acids; anodynes; tonics; salicylic acid and quinine, the last two chiefly from their antipyretic influence.

Carbolic acid and tincture of iodine, in doses of a minim each, repeated every two or three hours, have been said to produce remarkable effects from their antizymotic properties. This suggestion, also applicable to the treatment of diphtheria and all of the so-called "germ diseases," has met with some favor in Germany and England.

Before entering upon the fourth consideration, *the regulation of the temperature*, I will read a few extracts with regard to the prevailing death-rates at different places, and the decreased mortality wherever systematic antipyretie treatment has been followed in typhoid fever.

Referring to previous reports in the *Journal*, I find that Dr. Ernest Besnier's valuable statistics of the prevailing diseases in Paris, for ten years previous to 1877, give a death-rate of 21.31 per cent. in 16,000 cases of typhoid fever.

In the London fever hospitals in 1877 the average mortality was twenty per cent.

At Basle, under expectant methods, there were twenty-seven per cent. of deaths, and about the same ratio prevailed elsewhere in Europe.

In this country no statistics on a large scale have been prepared in any of the cities, but it is doubtful whether a sufficient number of cases from which to generalize would show a lower rate of mortality than fifteen per cent.

In England the number of deaths from typhoid fever annually is more than 8,000, and this disease is always an important factor in raising our death-rate, especially to be deplored as the victims have rarely reached the prime of life.

Therefore it is a serious question whether this high rate of mortality cannot be reduced materially in this country, as it has been in other countries.

It is now twenty years or more since Dr. Brand published his first monograph on the systematic treatment of typhoid fever by cold baths, acting on the supposition that the prolonged high temperature was the source of all degenerative changes; attacking the disease through its principal symptom. The zeal of this enthusiastic physician and his remarkable results were not sufficient, however, to popularize such a troublesome mode of treatment, and in his second work, published in 1868, he recognizes the fact that if his methods had not been adopted by Professor Bartels, of Kiel, they would have come to an early end. Later, Jürgensen, Liebermeister, Traube, and other German physicians of large opportunities took the matter up; after the war the same system was introduced successfully into the Lyons hospitals by medical officers who were forced to admit its merits after observing the practice of Dr. Brand on the French prisoners of war at Stettin; judging from the Croonian lectures of Dr. Cayley, delivered in 1880, it has slowly gained ground in London; but in this vicinity, with the exception of a small proportion of the cases at the Boston City Hospital, under the care of Drs. Edes, Stedman, Doe and Draper, it does not appear to have been tried.

Of course in a long disease, with constant calls upon the endurance of the attendants, useless labor is to be condemned, and proper conservatism inculcated.

Professor Gairdner, of Glasgow, where the mortality in the fever hospitals appears to be unusually low under the expectant plan, had a prolonged controversy with Professor Liebermeister on this subject.

In Professor Bamberger's wards in Vienna, in 1872, the results of antipyretic treatment were not striking, but in Berlin in the same winter Professor Traube thought that the mortality would be reduced to three per cent.

And, finally, Dr. Brand's last publication announces a death-rate of but 7.4 per cent. in a collection of 8,000 cases treated according to his methods in different parts of Europe.

Statistics are to a certain extent unreliable, but I think to only a moderate degree in this case; and as long as we have for some time been in the habit of accepting everything else that is German, the systematic antipyretic treatment of fevers, with all the attendant trouble and expense, should at length command a trial in hospital practice at least.

It may be said, I think, that the type of fever in Boston is not usually very severe. During the month of October and part of November last more than thirty cases came under my care at the City Hospital. Although their course was often tedious, with a very large proportion of relapses, two cases only were fatal. Several cases continued more than sixty days, and one case over one hundred days. Venous thrombosis affecting one or both legs was noticed in several instances. The relapses were not attributable to errors of diet, and bathing had not been resorted to except in the form of sprinkling or sponge baths.

In this connection the use of salicylate of soda *after* defervescence, as recommended by Professor Immermann, for the purpose of preventing relapses by disinfecting the system, is interesting.

The proper disposal of the stools is a matter of importance too often neglected.

Dr. Edes said that the lighter cases of typhoid, such as were the most of those which he had seen the change in his hospital service, needed but little treatment beyond ventilation, and some attention to diet. Quinia might be used as an antipyretic around the bed to hasten and make more complete the defervescence, but could hardly be considered essential. The cold sponging either with water or diluted alcohol contributed much to the comfort of the patient, and, so far as it had any influence upon the temperature, was in the right direction; but it could not be regarded as so efficient as some of the other methods of bathing.

In the severer cases he believed in the usefulness of antipyretic measures, especially the cold or gradually cooled bath. This is not supposed to shorten the disease, unless by diminishing the time of convalescence, but to render its whole course milder, especially if the treatment is begun early in the disease.

He referred to his own experience in three successive years in the City Hospital, though without claiming any special value for his own results, excepting as agreeing with those derived from a larger number of cases.

Some of the German statistics which had been already referred to by Dr. Mason, seemed to him valuable as showing the results in the change of methods of treatment in the same hospitals.

Even the table compiled by Bordier, who is not a strenuous advocate of this treatment, show a decided advantage on the side of bathing.

The labor involved is one of the important objections to the treatment, but he had not encountered so much opposition from attendants as he expected, and could not help thinking that the trouble of bathing was, in part at least, compensated for by the greater freedom from involuntary discharges, the care of which, in severe cases, constitutes a large and disagreeable part of the labor of the nurse.

Quinine is occasionally useful and very materially diminishes the number of baths necessary to keep down the temperature. Its effect is usually felt for many hours after the dose is given. Digitalis appeared to him inefficient in small doses and dangerous in large.

Dr. Tarbell said that the treatment of typhoid under his care in the wards of the Massachusetts General Hospital consisted in milk diet, sponge bathing with tepid water two or three times a day, or oftener if the temperature seemed to demand it, and abstinence from drugs. Beef tea was used as a stimulant in some cases, but alcoholic stimulants were used comparatively rarely and usually not at all until the third week or later in the disease. The diarrhœa was restrained from becoming excessive by means of small opiates.

Dr. Tarbell was exceedingly skeptical as to the utility of any of the so-called antipyretic remedies employed for rapidly and violently reducing the temperature, and he was not in the habit of giving large doses of quinine nor of using the cold tub bath. His experience was of course limited, but the results of treatment by these methods thus far published in England and America did not appear to demonstrate their superiority to the milder treatment. He also called attention to the comparative rarity of the propagation of the disease by direct contagion, and mentioned the fact that no nurse or other person had contracted the disease from a typhoid patient in the Massachusetts General Hospital for the past six years. The only means of prevention used were extreme cleanliness and the placing of carbolic acid in the bed-pans before and after using, which could at best be but a slight disinfection of the excreta which were then emptied into the common sewer.

Dr. C. E. Stedman said that he had reviewed for the forthcoming volume of the City Hospital Reports 1,032 cases of typhoid fever admitted in the last ten years; in the fifteen years since the opening of the Hospital 1,184 cases have been treated. The fatality in the first five years was thirteen per cent.; in the last ten years seventeen per cent. This mortality is about the same as that reported by Murchison in the London Fever Hospital from 1848 to 1870. It has been accounted for by the poor circumstances and constitutions of the patients, but the experience of the speaker confirmed the statement of Dr. Murchison that the fever was more fatal, in a course of years, in the higher than in the lower classes, and Dr. Stedman considered the high rate due to the fact, not only of so many moribund patients being received, but that the number was so large of patients admitted in the second and later weeks of the disease, not having had sufficient food. In one year the mortality was over twenty per cent., in another eight or nine per cent. In trying to tabulate the results of treatment, he had been freshly impressed with the untrustworthiness of such statistics. As well as it could be figured, the highest mortality was found among the patients treated by large doses of alcohol, but these were exactly the worst cases recorded in the books. Again, there were nearly four hundred cases which were set down as having had "no treatment," meaning that rest and milk were the chief or only restorative measures adopted. Of these the very mildest cases, seven per cent., died, being patients moribund on entrance who were incapable of receiving treatment, or such as were doing well till a sudden reverse took place. Eleven per cent. of those who had antipyretic doses of quinine, etc., died, nearly all bad cases; twelve per cent. of patients treated mainly by sponge baths succumbed, mostly mild types of fever. The calomel method recorded seven per cent. of mortality, but there were few cases thus treated. Of eighty patients treated by tub baths, twenty per cent. died, almost all bad cases. The naked figures unqualified by statement of the stage of the fever when admitted, or its severity, give little notion of the true standing of the facts. Dr. Stedman had faithfully used the antipyretic remedies, quinine, baths, etc., and found, like others, that such means reduced temperature in a marked degree; but the temperature almost always rose again, and he could not see that the course of the fever was at all shortened. It seemed to him that about six hundred of the cases above reported would have done well under expectant treatment, and of the remainder very many were saved by timely and proper medical supervision with the best of nursing. Skill in the handling of fever is attained when the physician knows that the time has come to supplement rest, food and nursing with the administration of drugs and stimuli. Dr. S. found the pulse was his best guide, and though knowing that patients with typhoid fever sometimes die with a slow pulse, had never himself yet lost a patient whose pulse did not range above 120. When the beats neared that number he began to feel anxious, and to stand ready to employ, if need be, the boldest stimulation. Records of temperature of 105° F. (in one 107° F.) had not alarmed him, if the pulse kept down. Several charts showed continued high temperatures with slight morning remissions, and a pulse of 100 or 108; such cases had done well. It is to be noticed that we saw few patients who had not been freely purged before admission, and he had been led to believe that a mild cathartic was not unwholesome early in the disease. For diarrhœa, when there were more than three or four stools daily, he used Dr. Harley's pill of a grain of opium and a quarter of a grain of sulphate of copper, or Dr. Pepper's pill of nitrate of silver and hyoscyamus. When stimulants did not moderate high delirium, chloral and bromide were serviceable, or sometimes the wet pack. For himself, were he limited to one remedy and one drug, he would cheerfully rely on cold sponging and brandy.

Dr. Minot had never employed cold baths in the treatment of typhoid fever, but he had tried the effect of sprinkling the patient with ice-water, aiding evaporation by fanning. In this way the temperature can be reduced several degrees in a short time, and the method is easy of application. He had, however, not found any great benefit from it in very grave cases. In two instances in which a high temperature was repeatedly lowered by the effusion, the patients died, but their condition was almost hopeless previously. The temperature can be reduced with much certainty by means of large doses of quinine, ten grains being given every fifteen minutes until the desired effect was obtained. Usually two or three doses are enough. He was accustomed to order stimulants freely in all cases with prostration, and he thought many patients were saved by them. He suggested a trial of the subcutaneous injection of sulphuric ether, as employed by Dr. L. E. Dupuy, of Paris.

Dr. Lyman said that the practical points in treatment of typhoid had been so much discussed by others that he would allude to only one or two.

In the first place, it was not a disease to be cured or cut short; a large proportion needed only nursing and watching, the physician to bear constantly in mind the old maxim of Cullen, "Obviate the tendency to death." It should be remembered always that it is the *heat* which kills, either directly or indirectly. True muscular degeneration, not simply muscular atrophy, it is which weakens the heart, and in a case which runs its normal course without complications it is this weakened heart which is, perhaps, more to be feared than anything else, a fact pointed out by Stokes as long ago as 1839. The difference between bodily heat and fever (quickened heart action as shown by the pulse, and the temperature of the blood as shown by the thermometer) should never be lost sight of. The necrobiotic action of heat upon the tissues, and the simple wasting from deficient nutrition are totally different things, the rocognition of which is necessary in our attempts at treatment.

He would say nothing as to cold bathing, having no experience of it, as he had always felt that he could accomplish with cold sponging, cooling drinks, quinine, and alcohol the diminution of dangerous heat without the liability to chill or local congestions.

He was in the habit of beginning with stimulants early, and was governed as to their continuance by the effect upon the pulse, skin and tongue. He thought it easier to forestall weakness of the heart than to remedy it when once established. As to quinine, he considered it our best and most reliable antiphlogistic; as an antipyretic its effect is more indirect, that is, by its property of lowering heat the tissue changes which cause fever are anticipated. This is the theory of excellent observers, and he was inclined to follow it. He wished also to remark upon the importance, in a prognostic point of view, of uniformity of both the pulse and the temperature, and especially the former; a steady pulse, for instance, of 120 being more favorable than a much lower but more irregular pulse.

Complications, of course, must be treated as such when they arise. Cerebral excitement, of course, counter-indicates quinine; a hard, full pulse, which, though not common, is sometimes found, would lead to caution in use of stimulants; exhausting diarrhœa would induce extra caution in the diet, and require either an alkali or gentle anodyne; much tympany, the use of turpentine, etc. Every case requires treatment, but in the majority of cases in his experience that treatment would be patience, diet, good nursing, while the few would, from complications or otherwise, require from beginning to end all the therapeutic skill of the most accomplished practitioner.

Dr. Mason said, in reply to a remark of Dr. Tarbell regarding the infrequency with which hospital attendants contract typhoid, that he remembered two cases in nurses in one year at the Massachusetts General Hospital, and several cases at the City Hospital. The poison which conveys the disease either *is* transmitted through the excreta or it is *not.* If it is, then disinfection of the stools is important, and for this purpose a strong solution of carbolic acid (one to twenty), or, better, a caustic solution of one of the mineral acids, is required. The practical difficulty of carrying out such measures, and of disinfecting other emanations from the body, was recognized, and Dr. Mason stated that he had seen two cases in which he was satisfied that the disease had been conveyed from one person to another by other means than the stools, perhaps by the breath.*

Dr. Reynolds remarked that the point of the propagation of typhoid fever through the fæcal discharges of patients is too important to be evaded or shuffled out of the way. It is conceivable that typhoid fever may have other ways of spreading itself. There is absolutely no question that the disease commucates itself by means of the dejections. The facts which incon-

---

* On subsequent inquiry of Dr. Rowe, superintendent of the City Hospital, it was found that in two and three-fourths years there had been four cases of typhoid among the attendants, three nurses and one ward tender, who had been on duty in the fever wards, where at one time last autumn there were sixty-nine patients with typhoid fever. He considered a solution of the substance sold under the name of "phenycle" a cheap and powerful disinfectant.

testably prove this have become classic in the literature of the subject. These facts are so well known that it is almost an impertinence to rehearse them. Two very striking instances are familiar to every reader of Liebermeister's essay.

" A woman attacked in Ulm returned to her native village, in which no case of typhoid had existed for many years. The excrements of this person were thrown upon a dunghill. Several weeks later five workmen were employed to remove this dunghill. Four of these five were attacked with typhoid, the fifth with gastric fever and swelling of the spleen. The excrements of these patients were buried deep in the dunghill. Nine months afterward the dunghill was wholly taken away by two men. One of these had typhoid, and died of it.

" In Lausen there had not been a case of typhoid for fifty-eight years. An epidemic of one hundred and thirty cases occurred, seventeen per cent. of the whole population. Only those who drank of the running streams were victims. Those who used the wells escaped. In the month preceding this outbreak, four cases of typhoid had developed in an outlying house, at some distance from Lausen. The excrements were thrown into a stream near the cottage. This stream communicated subterraneously with the streams that supplied Lausen."

The negative fact that nurses and others in close attendance upon fever patients do not contract the disease, provided due care is exercised in the disinfection of stools, in their instantaneous removal under proper precautions, and in burying them under dry earth, where that is practicable, is of much weight as to the matter under discussion.

Certain features of the disease, and some points in regard to its management aud its treatment by remedies, have been much impressed upon me in an epidemic of forty or fifty cases, which occurred in the neighborhood of Boston during the past summer. A large proportion of these cases were under my personal care.

I am anxious to urge in regard to arresting the spread of typhoid that the mild and imperfectly characterized cases are those most likely to do mischief. A servant, "neither sick nor well," whom the household are hardly willing to consider ill, school children suffering from the abortive form of the disease, these, and other like patients, deposit in the common privy excrements containing the poison, and later, if from any cause members of the family are in a susceptible condition, more than one life may be put in peril from attacks of which the real cause escapes recognition.

Twelve or fifteen years ago the writer of an exhaustive article on typhoid, in the *British and Foreign Medico-Chirurgical Journal*, laid stress upon a surmise that there exist in typhoid fever two stages: one an initial period, without general septic poison, and a following condition mainly characterized by symptoms which he believed to be due to septic absorption. He declares that this latter class of symptoms rarely appear, if at the commencement of the disease care be taken to secure sufficient unloading of the bowels, and if subsequently, by the continuance of mild laxatives and enemata, moderate daily dejections are obtained. I am inclined to admit at least a germ of truth in this statement.

The epidemic to which I have referred was essentially mild in type. It was treated under unusually favorable conditions as to season of the year, good local climate, and opportunity for careful nursing. Severe symptoms of various kinds were, however, in some cases present, giving ground for grave anxiety as to the issue. It seemed to me remarkable that in no one instance of those which I was permitted to watch was there intestinal hæmorrhage, diarrhœa, tympanites, or marked abdominal distress, and I could not help asking myself, whether pursuance of treatment like that just described might not have been in some degree responsible for this favorable result.

Not until we fairly admit the self-limited character of typhoid, the fact that the disease, once present, as inevitably runs its prescribed course as measles or scarlet fever do theirs, can we hope to understand it satisfactorily or to manage it wisely.

Those are golden words in Sir William Jenner's late lecture on the "Treatment of Typhoid," in which he describes the aggravation of symptoms which constantly follows the every-day futile attempt to fight off a threatened attack, sometimes by feeding and stimulants, sometimes by violent muscular exercise, and again by drugs, or pictures the mischief which often results from an imagined necessity of returning to a distant home to be ill.

